# Supplementary figures and images for: Dopamine increases protein synthesis in hippocampal neurons enabling dopamine-dependent LTP
Source: eLife. 2025 Mar 10;13:RP100822. doi: 10.7554/eLife.100822 (PMC11893101; doi:10.7554/eLife.100822)

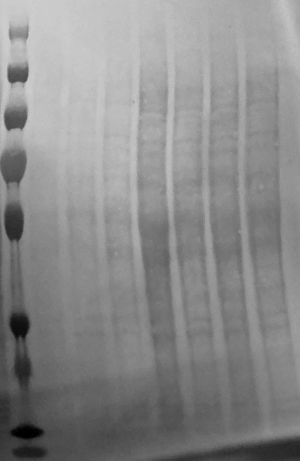

Supplement: Figure 1—source data 2. [file elife-100822-fig1-data2.zip › Figure 1 source data 2/ponceau.tif]

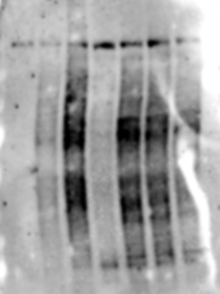

Supplement: Figure 1—source data 2. [file elife-100822-fig1-data2.zip › Figure 1 source data 2/puromycin.tif]

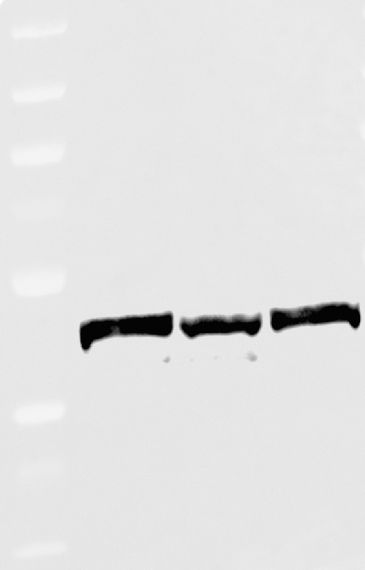

Supplement: Figure 3—source data 2. [file elife-100822-fig3-data2.zip › Figure 3 source data/Actin ladder.tif]

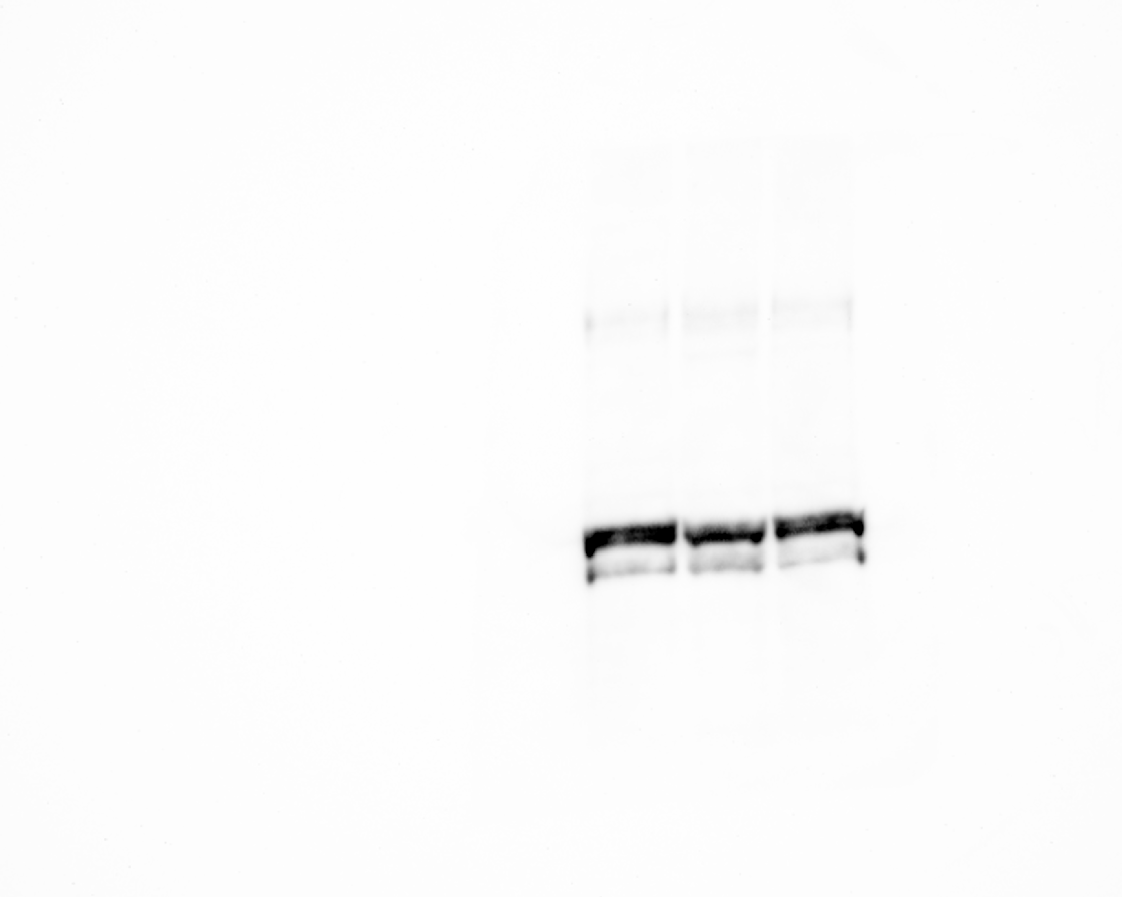

Supplement: Figure 3—source data 2. [file elife-100822-fig3-data2.zip › Figure 3 source data/actin.tif]

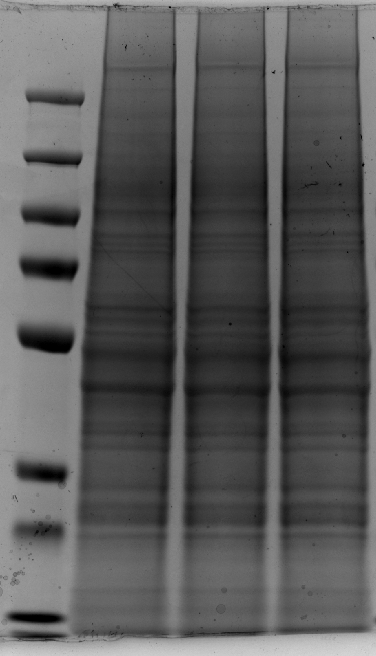

Supplement: Figure 3—source data 2. [file elife-100822-fig3-data2.zip › Figure 3 source data/Coomassie.tif]

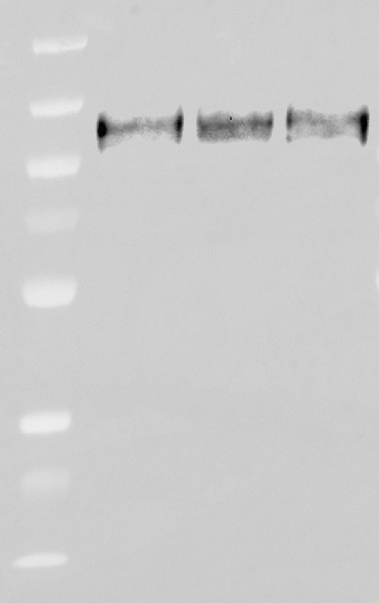

Supplement: Figure 3—source data 2. [file elife-100822-fig3-data2.zip › Figure 3 source data/Glua1 ladder.tif]

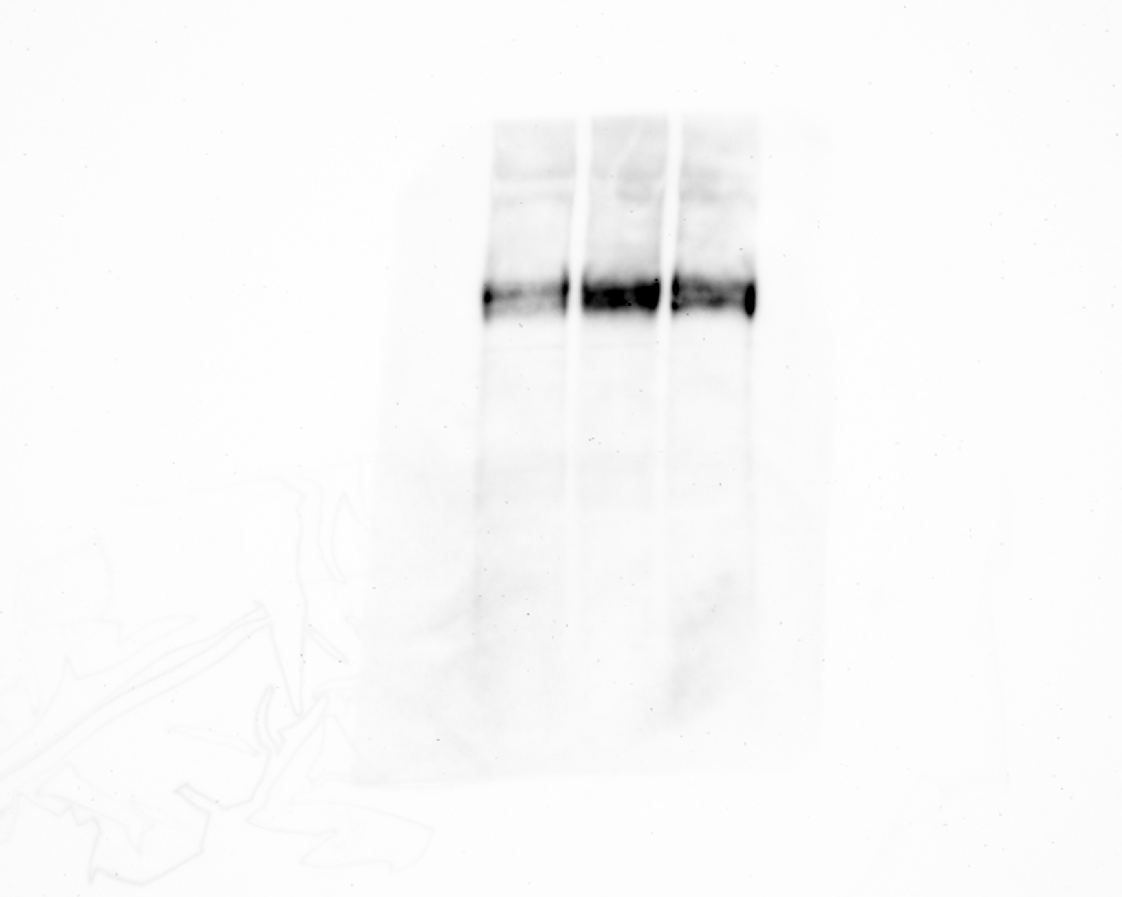

Supplement: Figure 3—source data 2. [file elife-100822-fig3-data2.zip › Figure 3 source data/glua1.tif]

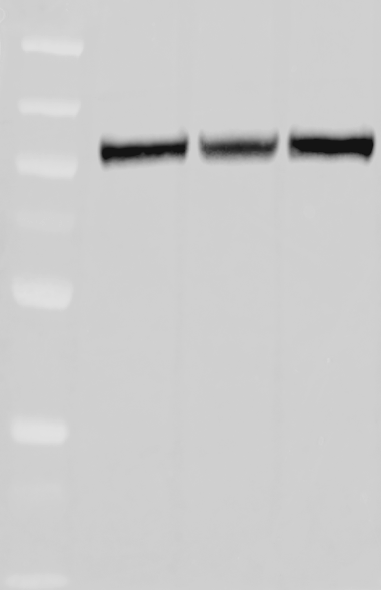

Supplement: Figure 3—source data 2. [file elife-100822-fig3-data2.zip › Figure 3 source data/Glua2 ladder.tif]

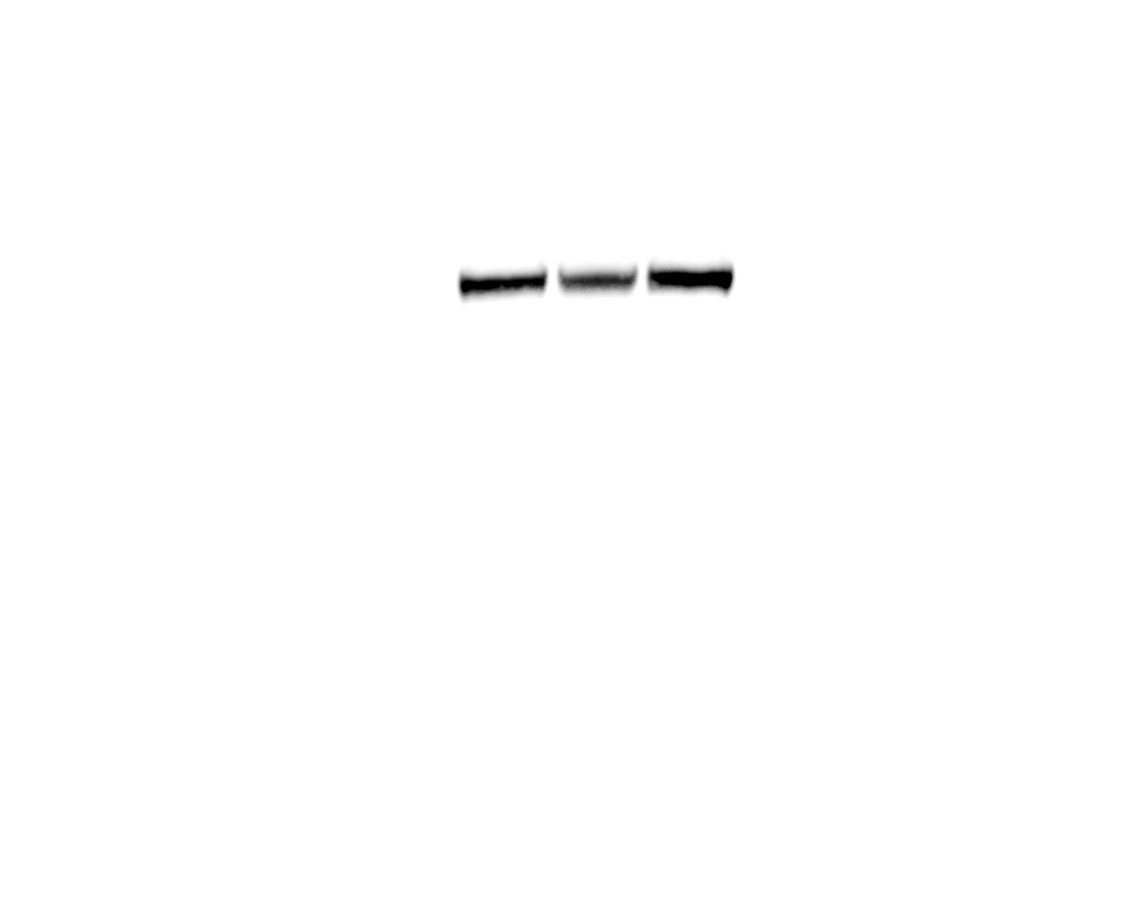

Supplement: Figure 3—source data 2. [file elife-100822-fig3-data2.zip › Figure 3 source data/glua2.tif]
