# Supplementary material for: Dopamine increases protein synthesis in hippocampal neurons enabling dopamine-dependent LTP
Source: eLife. 2025 Mar 10;13:RP100822. doi: 10.7554/eLife.100822 (PMC11893101; doi:10.7554/eLife.100822)
Supplement: Figure 1—source data 3. [file elife-100822-fig1-data3.pdf]

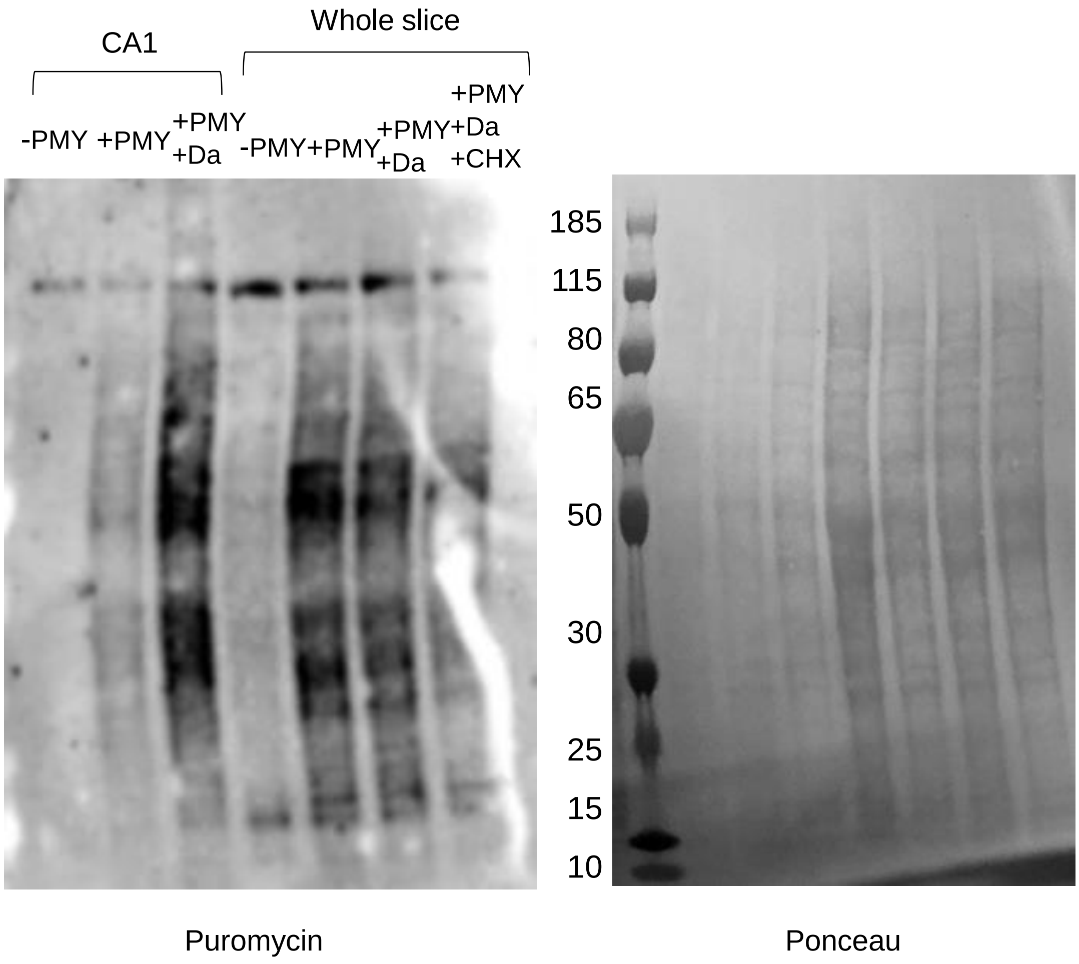

Figure 1, Source data 1. Original western blots corresponding to Figure 1D. Pageruler prestained ladder was used, with the molecular weights shown in kDa..
