# Supplementary material for: Dopamine increases protein synthesis in hippocampal neurons enabling dopamine-dependent LTP
Source: eLife. 2025 Mar 10;13:RP100822. doi: 10.7554/eLife.100822 (PMC11893101; doi:10.7554/eLife.100822)
Supplement: Figure 3—source data 3. [file elife-100822-fig3-data3.pdf]

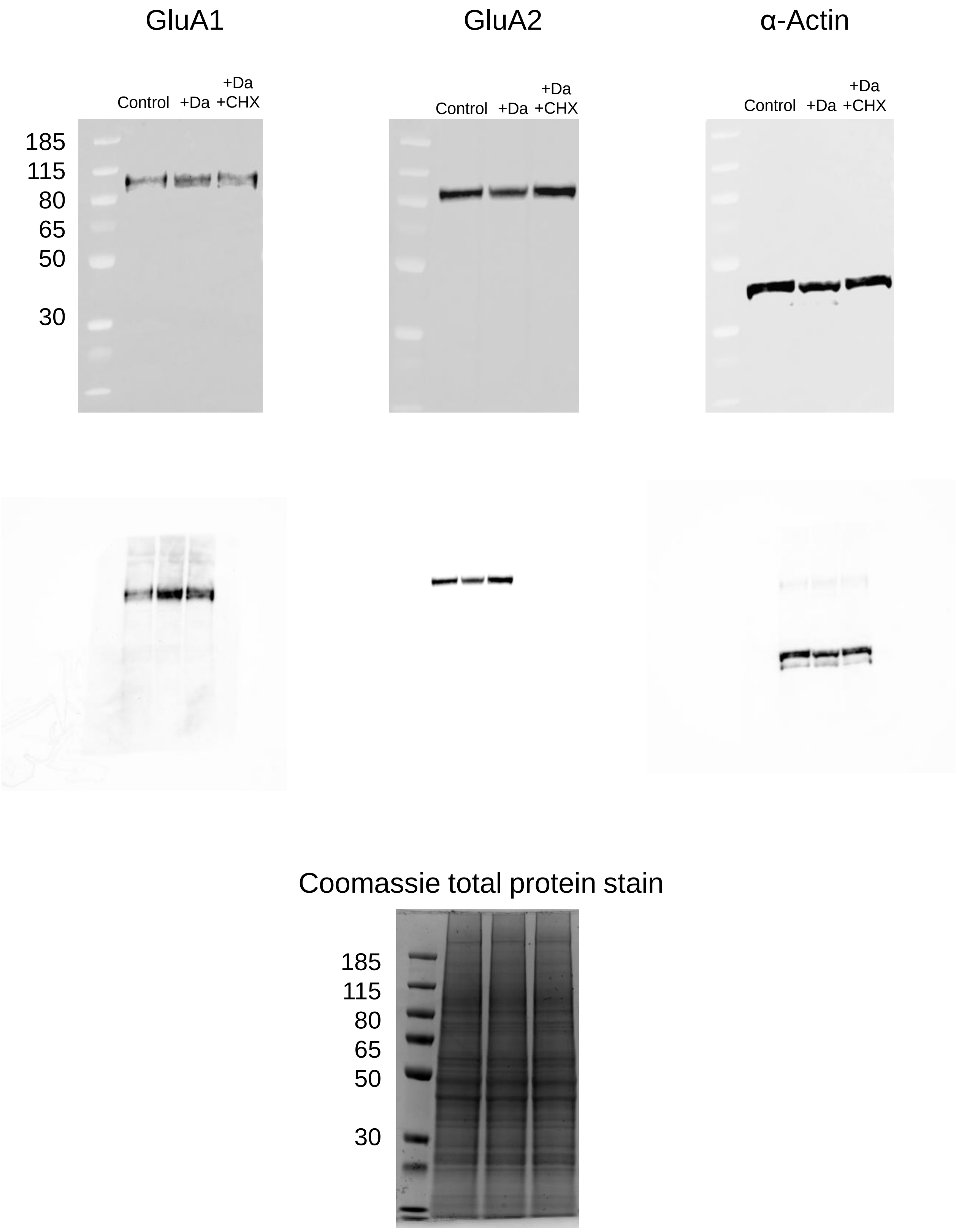

Figure 3, Source data 1. Original western blots shown both with and without ladder overlay corresponding to Figure 3A (GluA1) and figure 3C (GluA2) with α-actin control and the total protein Coomassie stain of the gel used for western blotting. Pageruler prestained ladder was used, with the molecular weights shown in kDa.
